# Supplementary material for: ZO-1 interacts with YB-1 in endothelial cells to regulate stress granule formation during angiogenesis
Source: Nat Commun. 2024 May 23;15:4405. doi: 10.1038/s41467-024-48852-7 (PMC11116412; doi:10.1038/s41467-024-48852-7)
Supplement: Supplementary file 1 — Supplementary Information [file 41467_2024_48852_MOESM1_ESM.pdf]

Figure S1

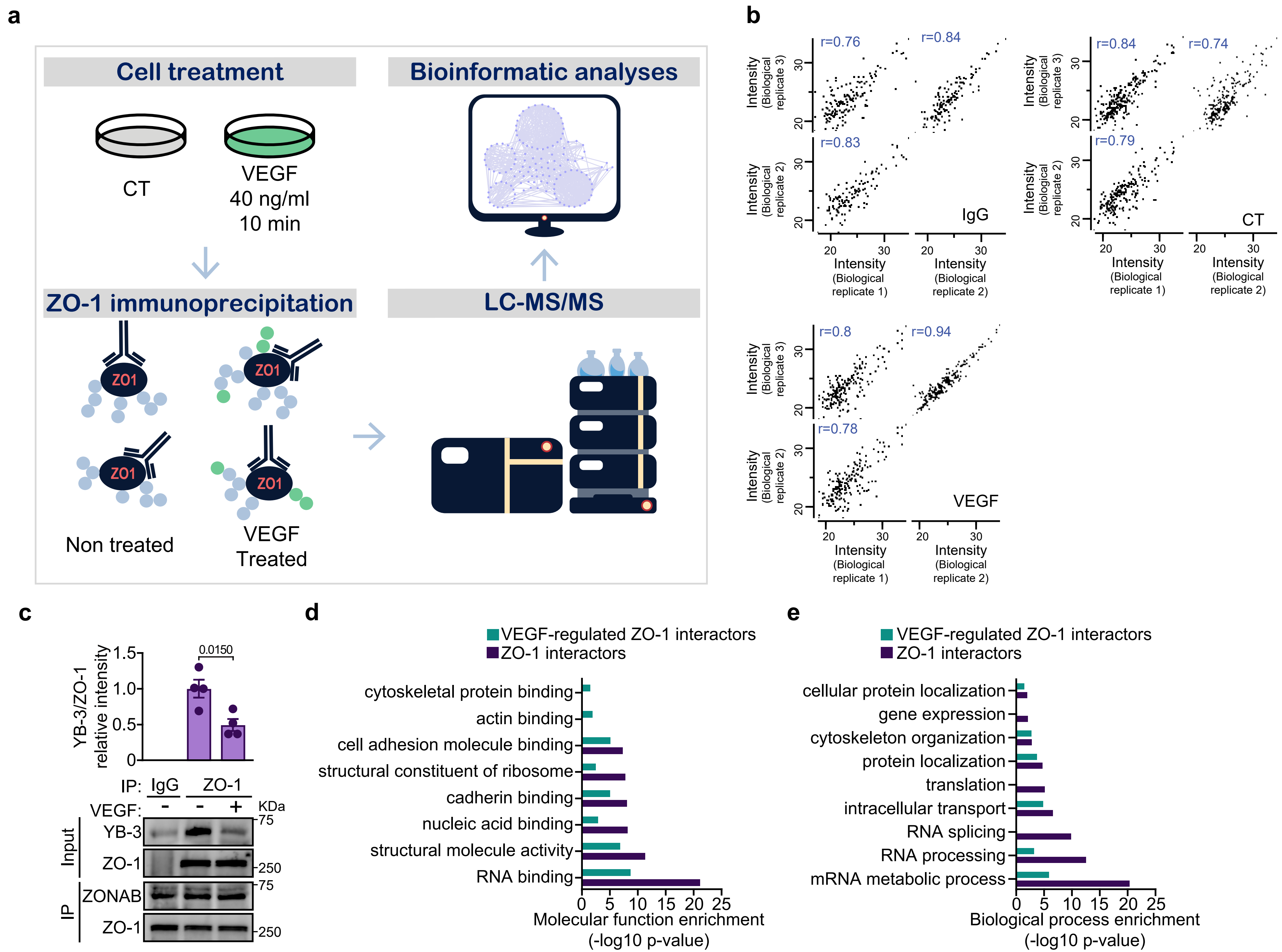
a
Cell treatment
CT
VEGF
40 ng/ml
10 min
Bioinformatic analyses
ZO-1 immunoprecipitation
LC-MS/MS
ZO1
Non treated
VEGF
Treated
b
r=0.76
r=0.84
r=0.83
IgG
r=0.84
r=0.74
r=0.79
CT
r=0.8
r=0.94
r=0.78
VEGF
Intensity (Biological replicate 1)
Intensity (Biological replicate 2)
Intensity (Biological replicate 3)
c
YB-3/ZO-1 relative intensity
0.0150
IP: IgG ZO-1
VEGF: - - +
KDa
Input
YB-3
ZO-1
IP
ZONAB
ZO-1
75
250
d
VEGF-regulated ZO-1 interactors
ZO-1 interactors
cytoskeletal protein binding
actin binding
cell adhesion molecule binding
structural constituent of ribosome
cadherin binding
nucleic acid binding
structural molecule activity
RNA binding
Molecular function enrichment (-log10 p-value)
e
VEGF-regulated ZO-1 interactors
ZO-1 interactors
cellular protein localization
gene expression
cytoskeleton organization
protein localization
translation
intracellular transport
RNA splicing
RNA processing
mRNA metabolic process
Biological process enrichment (-log10 p-value)

Figure S1: Proteomic profiling of the ZO-1 interactome in ECs.

a, Overview of the experimental proteomics pipeline: BAECs were treated with VEGF (40 ng/ml, 10 min) and endogenous ZO-1 was immunoprecipitated using antibodies against ZO-1 (1 μg). Immunoprecipitates were processed by LC-MS/MS and bioinformatics analyses were performed as indicated in materials and methods. **b,** Correlation scatter plots of peptide intensities between biological replicates in control immunoprecipitations (IgG), untreated (CT) and VEGF-treated cells. **c**, Co-immunoprecipitation of ZONAB and ZO-1 from BAEC lysates treated or not with VEGF (40 ng/ml, 10 min). Non-immune IgG serves as control for non-specific co-immunoprecipitation. Levels of immunoprecipitated proteins were determined by immunoblot using the indicated antibodies. Histogram showing quantification of the ratio of ZONAB levels relative to ZO-1 (n = 4 independent experiments). Unpaired two-tailed Student's *t* test. Data are represented as mean values ± SEM. **d, e**, Enrichment analysis for GO molecular function (**d**) and biological process (**e**) terms of ZO-1 interacting proteins.

Figure S2

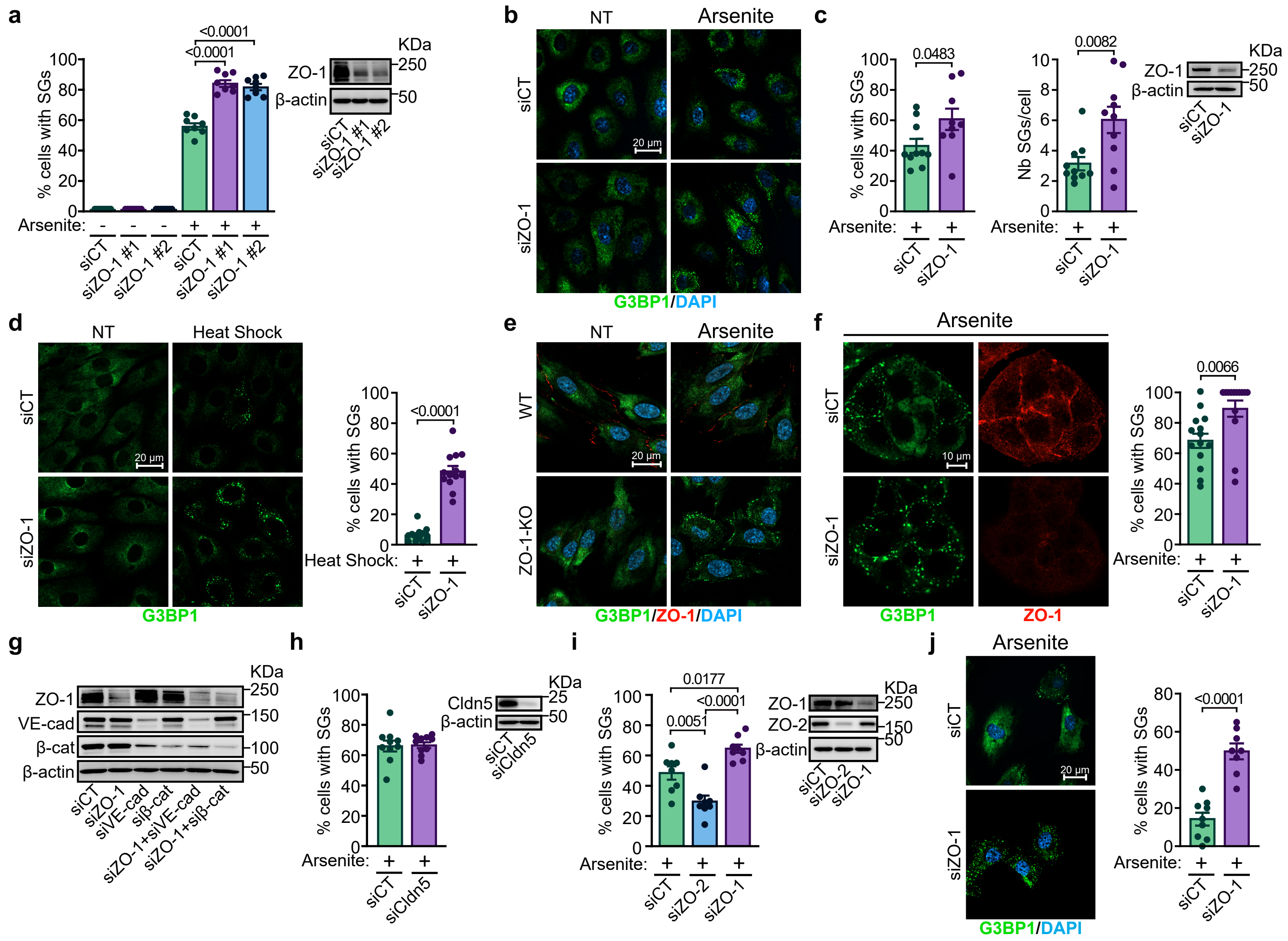
a
% cells with SGs
<0.0001
Arsenite:
siCT
siZO-1 #1
siZO-1 #2
ZO-1
β-actin
KDa
250
50
b
NT
Arsenite
siCT
siZO-1
20 µm
G3BP1/DAPI
c
0.0483
0.0082
Nb SGs/cell
d
Heat Shock
G3BP1
e
WT
ZO-1-KO
G3BP1/ZO-1/DAPI
f
10 µm
ZO-1
0.0066
g
VE-cad
β-cat
siVE-cad
siβ-cat
siZO-1+siVE-cad
siZO-1+siβ-cat
150
100
h
Cldn5
siCldn5
25
i
0.0177
0.0051
ZO-2
siZO-2
j
0.0001

Figure S2: ZO-1 depletion increases SG formation in ECs.

a, Downregulation of ZO-1 using two different siRNA increased SG formation. Quantification of G3BP1 positive SGs of immunofluorescence staining of SGs using antibodies against G3BP1 in HUVECs transfected with siCT and siZO-1 #1 or siZO-1 #2 in absence or presence of sodium arsenite (500 μM for 30 min). The percentage of cells with SGs was calculated in n = 8 fields of view per condition and at least 25 cells/field. One-way ANOVA followed by Bonferroni's multiple comparison tests. The downregulation efficiency of siRNA was confirmed by immunoblot (inset). **b,** Increased SG formation in ZO-1 depleted BAECs. Immunofluorescence staining of SGs using antibodies against G3BP1 in siCT and siZO-1 transfected BAECs in absence (NT) or presence of arsenite (500 μM for 30 min). Nuclei are stained with DAPI. **c,** Quantification of G3BP1 positive SGs shown in (**b**). The percentage of cells with SGs (left) or the number of SGs per cell (right) was calculated in n = 10 fields of view per condition and at least 15 cells/field. Unpaired two-tailed Student's *t* test. The downregulation efficiency of siRNA was confirmed by immunoblot (inset). **d,** Increased SG formation in ZO-1 depleted ECs in response to heat shock. Immunofluorescence staining of SGs using antibodies against G3BP1 in siCT and siZO-1 transfected BAECs exposed or not to heat shock (42°C for 2 h). The percentage of cells with G3BP1 positive SGs was calculated in n = 13 fields of view per condition and at least 15 cells/field. Unpaired two-tailed Student's *t* test. **e,** Increased SG formation in CRISPR/Cas9-generated ZO-1 knockout TeloHAECs. Immunofluorescence staining of SGs using antibodies against G3BP1 wildtype (WT) or ZO-1 knockout (ZO-1-KO) TeloHAECs in absence (NT) or presence of sodium arsenite (500 μM for 30 min). Nuclei are stained with DAPI. **f,** Increased SG formation in ZO-1-depleted HepG2 cells treated with arsenite. HepG2 cells transfected with siZO-1 or siCT were treated with sodium arsenite (500 μM for 30 min) and stained by immunofluorescence for SGs using antibodies against G3BP1 (green) and for ZO-1 (red). The percentage of cells with G3BP1 positive SGs was

quantified in n = 14 fields of view per condition and at least 15 cells/field. Unpaired two-tailed Student's *t* test. **g,** Immunoblots to document the efficiency of siRNAs against ZO-1, VE-cadherin, β-catenin, ZO-1 and VE-cadherin or ZO-1 and β-catenin shown in Figure 2d. Note that in ECs downregulation of VE-cadherin is known to also affect β-catenin levels. **h,** Downregulation of claudin-5 does not increase the number of SGs. HUVECs transfected with siCT or siRNA against claudin-5 (siCldn5) were treated with sodium arsenite (500 μM for 30 min). The percentage of cells with G3BP1 positive SGs was calculated in n = 10 fields of view per condition and at least 25 cells/field. Unpaired two-tailed Student's *t* test. The downregulation efficiency of siCldn5 was confirmed by western blot (inset). **i,** Downregulation of ZO-2 does not increase SG formation in response to arsenite. BAECs transfected with siCT, siRNA against ZO-2 (siZO-2) or siZO-1 were treated with sodium arsenite (500 μM for 30 min). The percentage of cells G3BP1 positive SGs was calculated in n = 8 fields of view per condition and at least 30 cells/field. One-way ANOVA followed by Bonferroni's multiple comparison tests. The downregulation efficiency of siZO-2 and siZO-1 was confirmed by western blot (inset). **j,** Downregulation of ZO-1 increases SG formation in sparsely plated BAECs. Immunofluorescence staining of SGs using antibodies against G3BP1 in sparsely plated and siCT or siZO-1-transfected BAECs in presence of arsenite (125 μM for 30 min). The percentage of cells with SGs was calculated in siCT n = 9 and siZO-1 n = 8 fields of view per condition and at least 10 cells/field. Unpaired two-tailed Student's *t* test. Data are presented as mean values ±SEM. Source data are provided in the Source Data file.

Figure S3

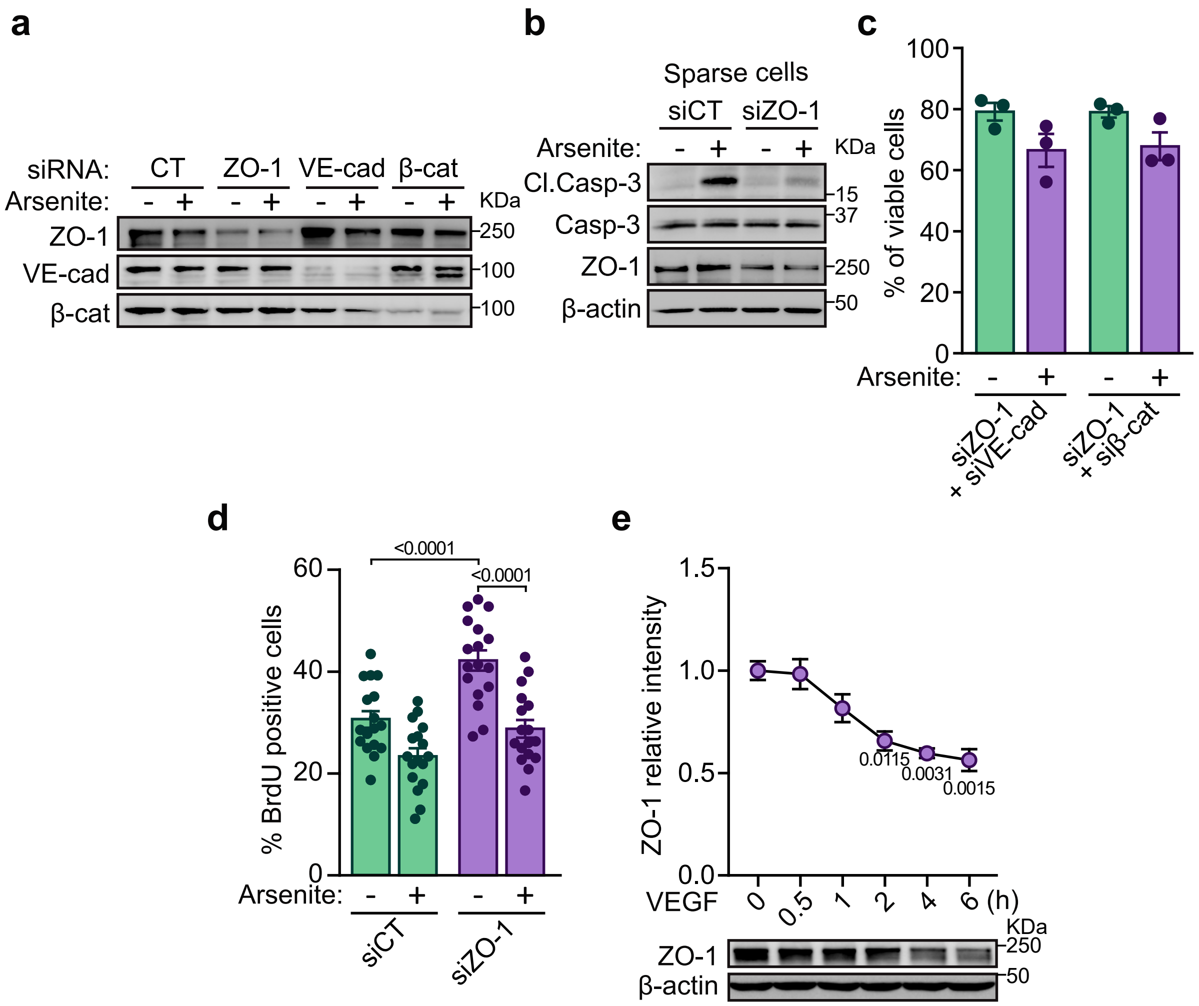
a
siRNA: CT ZO-1 VE-cad β-cat
Arsenite: - + - + - + - +
KDa
ZO-1
VE-cad
β-cat
250
100
100
b
Sparse cells
siCT siZO-1
Arsenite: - + - +
KDa
Cl.Casp-3
Casp-3
ZO-1
β-actin
15
37
250
50
c
% of viable cells
100
80
60
40
20
0
Arsenite: - + - +
siZO-1 + siVE-cad
siZO-1 + siβ-cat
d
% BrdU positive cells
60
40
20
0
<0.0001
<0.0001
Arsenite: - + - +
siCT
siZO-1
e
ZO-1 relative intensity
1.5
1.0
0.5
0.0
0.0115
0.0031
0.0015
VEGF 0 0.5 1 2 4 6 (h)
KDa
ZO-1
β-actin
250
50

Figure S3: ZO-1 depletion increases viability of ECs.

a, Immunoblots to document the efficiencies of siRNAs against ZO-1, VE-cadherin or siβ-catenin shown in Figure 4b. Note that downregulation of VE-cadherin is known to also affect β-catenin levels in ECs. **b,** Representative immunoblot analysis of cleaved caspase-3 and total caspase-3 in siCT and siZO-1 transfected and sparsely plated BAECs, and in presence or not of arsenite (50 μM; 6 h). **c**, Cell viability, measured by Trypan blue exclusion, of HUVECs transfected with a combination of siRNAs against ZO-1 and VE-cadherin or ZO-1 and β-catenin and treated or not with arsenite (25 μM; 12 h) (n = 3 independent experiments). **d,** Quantification of BrdU incorporation in siCT and siZO-1-transfected HUVECs in absence or presence of sodium arsenite (500 μM for 30 min) (n = 17 fields of view per condition and at least 25 cells/field). **e**, Kinetics of ZO-1 expression levels in HUVECs treated with VEGF (40 ng/ml). Protein expression levels were determined by immunoblot. β-actin serves as a loading control (n = 3 independent experiments). Comparisons of basal expression levels versus treated conditions were performed for each. (**c-e**) One-way ANOVA followed by Bonferroni's multiple comparison tests. Data are presented as mean values ± SEM. Source data are provided in the Source Data file.

Figure S4

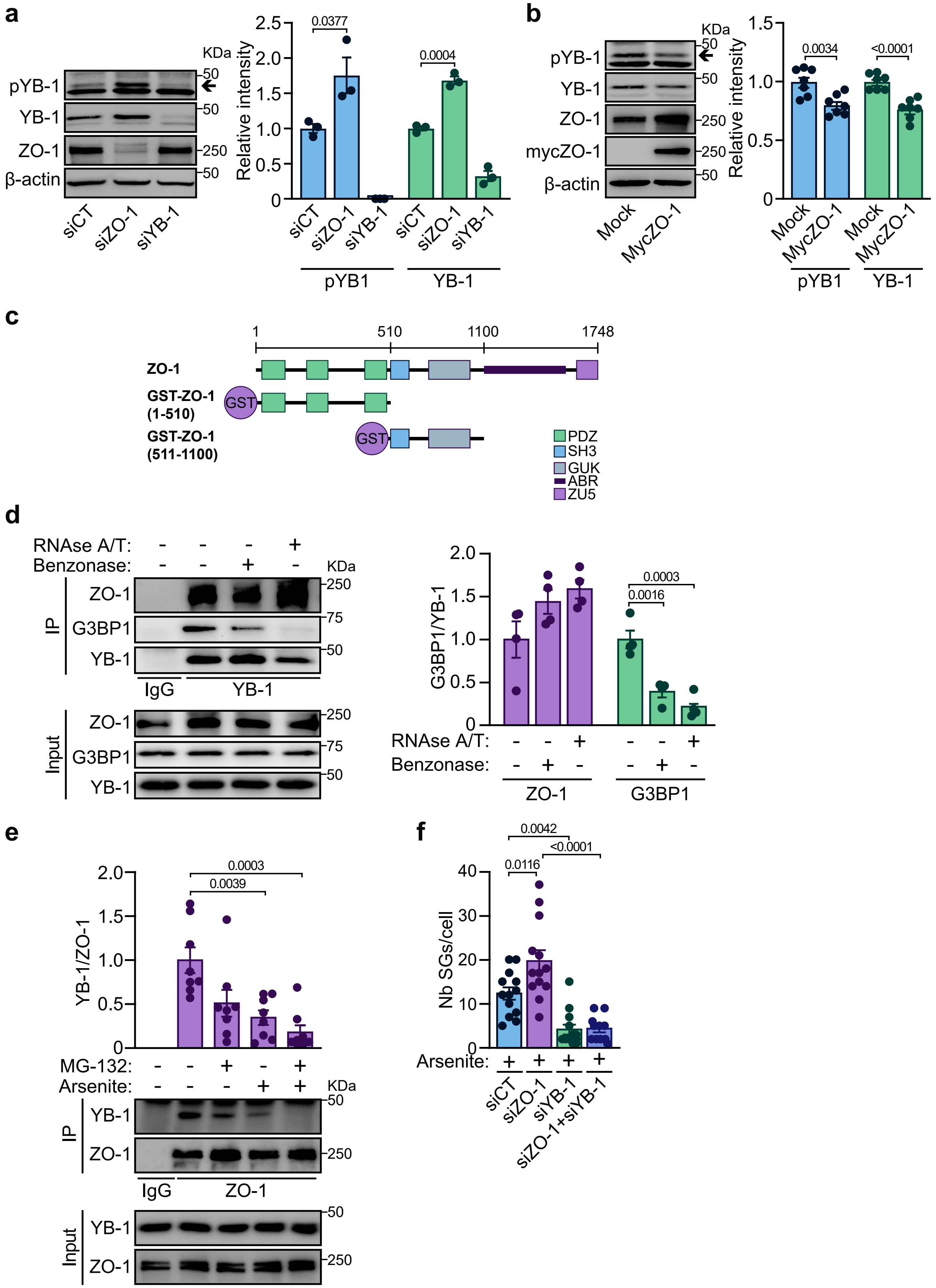

a
KDa
pYB-1
YB-1
ZO-1
β-actin
50
50
250
50
siCT
siZO-1
siYB-1
Relative intensity
0.0377
0.0004
pYB1
YB-1
b
pYB-1
YB-1
ZO-1
mycZO-1
β-actin
Mock
MycZO-1
0.0034
<0.0001
c
1
510
1100
1748
ZO-1
GST-ZO-1 (1-510)
GST-ZO-1 (511-1100)
GST
PDZ
SH3
GUK
ABR
ZU5
d
RNAse A/T:
Benzonase:
IP
Input
ZO-1
G3BP1
YB-1
IgG
G3BP1/YB-1
0.0003
0.0016
e
YB-1/ZO-1
MG-132:
Arsenite:
f
Nb SGs/cell
0.0042
<0.0001
0.0116
siZO-1+siYB-1

Figure S4: The binding between ZO-1 and YB-1 is not RNA dependent and ZO-1 levels modulate YB-1 phosphorylation.

a, Increased YB-1 phosphorylation in ZO-1 depleted cells. Immunoblot analysis of pYB-1 and YB-1 in BAECs transfected with siCT, siZO-1 or siYB-1. Histogram referring to the quantification of pYB-1 and YB-1 levels relative to β-actin in three independent experiments. **b,** Overexpression of ZO-1 decreases YB-1 phosphorylation. Immunoblot analysis of pYB-1 and YB-1 in BAECs expressing myc-tagged ZO-1 (mycZO-1). Histogram referring to the quantification of pYB-1 and YB-1 levels relative to β-actin (n = 7 independent experiments). Unpaired two-tailed Student's *t* test. **c,** Schematic representation of the domain organizations of the ZO-1 protein sequence and of GST fusion ZO-1 constructs, GST-ZO-1 (aa. 1-510) and GST-ZO-1 (aa. 511-1100) used for GST pulldown experiments. Structural domains are denoted by colors and annotated. **d,** The association between ZO-1 and YB-1 is not dependent on RNA. YB-1 was immunoprecipitated from BAECs lysates and samples were treated or not with RNAse A/T (0.5µl/ml, 10 min at 37 °C) or benzonase (1µl/ml, 30 min at 4 °C). Presence of ZO-1 or G3BP1 in the YB-1 immunoprecipitates were determined by immunoblot. Non-immune IgG serves as control for immunoprecipitation. Histograms show the quantification of the ratio of G3BP1 or ZO-1 levels relative to YB-1 present in the immunoprecipitates (n = 4 independent experiments). **e,** Inhibition of the proteasomal degradation of ZO-1 does not affect the ZO-1/YB-1 interaction. ZO-1 was immunoprecipitated from lysates of BAECs treated or not with MG132 (40 µM; 60 min) and then with arsenite (25 µM; 30 min). Presence of YB-1 in the ZO-1 immunoprecipitates was determined by immunoblot. Non-immune IgG serves as control for immunoprecipitation. Bar graph shows the ratio of YB-1 levels relative to ZO-1 present in the immunoprecipitates (n = 8 independent experiments). **f,** Histogram showing the number of SGs per cell referring to the immunofluorescence in Fig. 5e. siCT, siZO-1,

siYB-1 n = 13; siZO-1 + siYB-1 n = 11 fields of view per condition and at least 10 cells/field. (**a, d-f**) One-way ANOVA followed by Bonferroni's multiple comparison tests. Data are represented as mean values ±SEM. Source data are provided in the Source Data file.

Figure S5

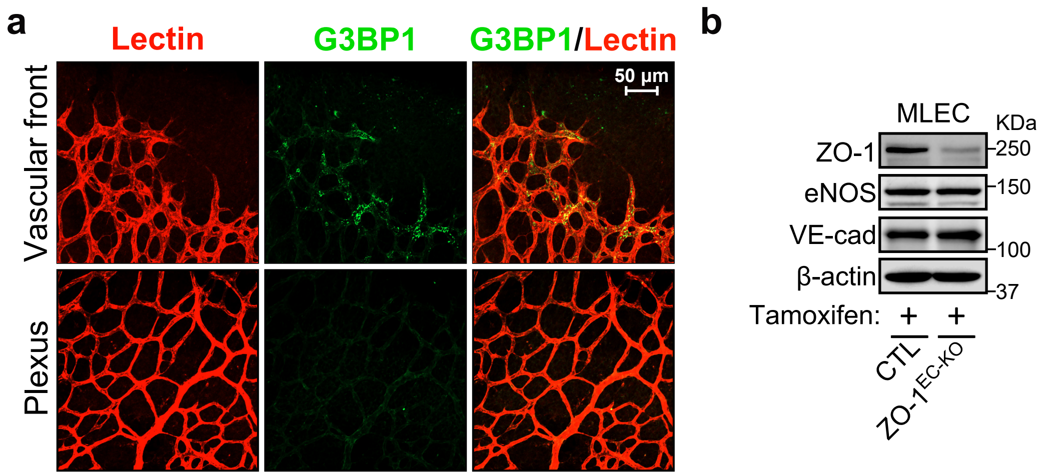
a
Lectin
G3BP1
G3BP1/Lectin
50 µm
Vascular front
Plexus
b
MLEC
KDa
ZO-1
eNOS
VE-cad
β-actin
250
150
100
37
Tamoxifen:
+
+
CTL
ZO-1EC-KO

Figure S5

a, Increased G3BP1 positive SGs in ECs of the vascular front of the developing retinal vasculature. SGs were stained in ECs of the vascular front (edge) and plexus (central) portions of the developing retinal vasculature of mice at P7 using antibodies against G3BP1 (green). ECs of the retinas were stained with Isolectin B4 (red). **b,** Mouse lung ECs (MLECs) from EC-specific and tamoxifen-inducible ZO-1 knockout mice. MLECs were isolated from lungs of Pdgfb-iCreER;Tjp1$^{fl/fl}$ (ZO-1$^{EC\text{-}KO}$) and from Tjp1$^{fl/fl}$ control mice (CTL) using mouse CD102 antibodies conjugated to magnetic Dynabeads. Isolated cells were grown in culture for ten to fourteen days. MLECs from both mouse lines were seeded at 50% confluence and treated with 4-hydroxytamoxifen (TAM; 100 nM) for 48 h. ZO-1 expression was monitored by Western blot. Blots against VE-cadherin, eNOS and β-actin serve as controls. Experiments were repeated using three mice for each genotype with identical results.
