# Supplementary material for: ZO-1 interacts with YB-1 in endothelial cells to regulate stress granule formation during angiogenesis
Source: Nat Commun. 2024 May 23;15:4405. doi: 10.1038/s41467-024-48852-7 (PMC11116412; doi:10.1038/s41467-024-48852-7)
Supplement: Supplementary file 3 — Description of Additional Supplementary Files [file 41467_2024_48852_MOESM3_ESM.pdf]

File name: Supplementary Data 1
Description: ZO-1 interactome analysis. List of ZO-1-interacting proteins identified by mass spectrometry and subjected to gene ontology enrichment analysis. Raw data of mass spectrometry analysis related to Figure 1 and S1.

File name: Supplementary Data 2
Description: Comparative analysis of ZO-1 and b-catenin interactome identified by mass spectrometry. Sheet-1: A comparison between ZO-1 and b-catenin interacting proteins identified by MS. Overlapping proteins were highlighted in Red. Sheet-2: Comparative list of RBPs identified in ZO-1 or b-catenin interactome. Overlapping proteins were highlighted in Red.

File name: Supplementary Data 3
Description: Cross-reference analysis between ZO-1 interactors and the CRAPome database. Sheet-1: The first column of the table shows the list of CRAPome identified in control experiments. Only experiments using agarose affinity support were used. The second column lists the averaged spectral counts for the selected protein across the experiments in which it was identified. The third column details the number of experiments in the database where the selected protein was detected (with at least one peptide having PeptideProphet probability of 0.9 or higher). Columns D to N listed the spectral counts in each of the control experiments in the CRAPome. Sheet-2: The first column of the table shows the list of entries submitted to CRAPome Web interface. The second column lists the averaged spectral counts for the selected protein across the experiments in which it was identified. The third column details the number of experiments in the database where the selected protein was detected (with at least one peptide having PeptideProphet probability of 0.9 or higher).
