# Supplementary material for: ZO-1 interacts with YB-1 in endothelial cells to regulate stress granule formation during angiogenesis
Source: Nat Commun. 2024 May 23;15:4405. doi: 10.1038/s41467-024-48852-7 (PMC11116412; doi:10.1038/s41467-024-48852-7)
Supplement: Supplementary file 7 — Reporting Summary [file 41467_2024_48852_MOESM7_ESM.pdf]

nature portfolio

Corresponding author(s): Jean-Philippe Gratton

Last updated by author(s): Apr 17, 2024

Reporting Summary

Nature Portfolio wishes to improve the reproducibility of the work that we publish. This form provides structure for consistency and transparency in reporting. For further information on Nature Portfolio policies, see our Editorial Policies and the Editorial Policy Checklist.

Statistics

For all statistical analyses, confirm that the following items are present in the figure legend, table legend, main text, or Methods section.

n/a	Confirmed	
☐	☒	The exact sample size (*n*) for each experimental group/condition, given as a discrete number and unit of measurement
☐	☒	A statement on whether measurements were taken from distinct samples or whether the same sample was measured repeatedly
☐	☒	The statistical test(s) used AND whether they are one- or two-sided<br>*Only common tests should be described solely by name; describe more complex techniques in the Methods section.*
☐	☒	A description of all covariates tested
☐	☒	A description of any assumptions or corrections, such as tests of normality and adjustment for multiple comparisons
☐	☒	A full description of the statistical parameters including central tendency (e.g. means) or other basic estimates (e.g. regression coefficient) AND variation (e.g. standard deviation) or associated estimates of uncertainty (e.g. confidence intervals)
☐	☒	For null hypothesis testing, the test statistic (e.g. *F*, *t*, *r*) with confidence intervals, effect sizes, degrees of freedom and *P* value noted<br>*Give P values as exact values whenever suitable.*
☒	☐	For Bayesian analysis, information on the choice of priors and Markov chain Monte Carlo settings
☒	☐	For hierarchical and complex designs, identification of the appropriate level for tests and full reporting of outcomes
☐	☒	Estimates of effect sizes (e.g. Cohen's *d*, Pearson's *r*), indicating how they were calculated

Our web collection on statistics for biologists contains articles on many of the points above.

Software and code

Policy information about availability of computer code

Data collection	Raw mass spectrometry data were processed using the MaxQuant software (version 1.5.3.17).<br>Database searching was performed using the Andromeda search engine (version 1.5.3.17).
Data analysis	Statistical analysis of MaxQuant output was done using Perseus (version 1.5.2.6.)<br>Zen Blue software (version 2.6)(Zeiss)<br>ImageJ (version 1.53)<br>Adobe Photoshop 2020 and 2022<br>Adobe Illustrator 2020 and 2022<br>Images of retinas were analysed using AngioTool (version 0.6)<br>Image Quant LAS4000<br>Graphpad Prism (version 6)<br>Data analysis and visualization of publicly available scRNA-sequencing data from P6 mouse retinas (GSE175895) was done using tools from Seurat R-package (version 3.2.3)

For manuscripts utilizing custom algorithms or software that are central to the research but not yet described in published literature, software must be made available to editors and reviewers. We strongly encourage code deposition in a community repository (e.g. GitHub). See the Nature Portfolio guidelines for submitting code & software for further information.

Data

Policy information about availability of data

All manuscripts must include a data availability statement. This statement should provide the following information, where applicable:

- Accession codes, unique identifiers, or web links for publicly available datasets
- A description of any restrictions on data availability
- For clinical datasets or third party data, please ensure that the statement adheres to our policy

The mass spectrometry proteomics data have been deposited to the ProteomeXchange Consortium via the PRIDE partner repository with the dataset identifier PXD02933283. STRING database version 11 was used to create all networks containing 24'584'628 proteins from 5090 organisms with 3'123'056'667 interactions which can be accessed via https://version-11-0.string-db.org/. Source data are provided with this paper.

Research involving human participants, their data, or biological material

Policy information about studies with human participants or human data. See also policy information about sex, gender (identity/presentation), and sexual orientation and race, ethnicity and racism.

Reporting on sex and gender	n/a
Reporting on race, ethnicity, or other socially relevant groupings	n/a
Population characteristics	n/a
Recruitment	n/a
Ethics oversight	n/a

Note that full information on the approval of the study protocol must also be provided in the manuscript.

Field-specific reporting

Please select the one below that is the best fit for your research. If you are not sure, read the appropriate sections before making your selection.

☒ Life sciences ☐ Behavioural & social sciences ☐ Ecological, evolutionary & environmental sciences

For a reference copy of the document with all sections, see nature.com/documents/nr-reporting-summary-flat.pdf

Life sciences study design

All studies must disclose on these points even when the disclosure is negative.

Sample size	The sample size for each in vitro experiment is detailed in the Figure Legends and Materials and Methods section. Each experiment was performed using at least three independent biological replicates. No explicit power analysis was conducted to pre-determine sample size. In general, sample size of cellular experiments was determined based on standards for experimental cell biology, attempting to have a minimum of n = 3 biological replicates with sufficient reproducibility. For mouse model experiments, depending on sample availability, sample size of minimum n=3 was used in the study. All attempts at data replication were successful. All sample sizes were listed in the corresponding figure legend or figures.
Data exclusions	No data exclusions in this manuscript.
Replication	For proteomics experiment three independent biological replicates and two technical replicates were performed.<br>The in vitro experiments were repeated independently at least three times, as indicated in the Figure Legends.
Randomization	Randomization is not relevant for this study because the mice are treated under the same conditions, i.e. at the same age with the same treatment. For cell line-based experiments, randomization was not required because all samples were analyzed equally.
Blinding	The investigators were blinded to group allocations since all animal experiments were done before the genotyping of the mice.<br>The investigators were blinded during ex vivo data analysis of the retinal vasculature. Furthermore, measurements were performed in an unbiased manner with softwares (ImageJ, Zen, AngioTool).

Reporting for specific materials, systems and methods

We require information from authors about some types of materials, experimental systems and methods used in many studies. Here, indicate whether each material, system or method listed is relevant to your study. If you are not sure if a list item applies to your research, read the appropriate section before selecting a response.

Materials & experimental systems

n/a	Involved in the study
☐	☒ Antibodies
☐	☒ Eukaryotic cell lines
☒	☐ Palaeontology and archaeology
☐	☒ Animals and other organisms
☒	☐ Clinical data
☒	☐ Dual use research of concern
☒	☐ Plants

Methods

n/a	Involved in the study
☒	☐ ChIP-seq
☒	☐ Flow cytometry
☒	☐ MRI-based neuroimaging

Antibodies

Antibodies used

Antibodies used for immunoprecipitation, immunoblots and/or immunofluorescence experiments:
Primary antibodies from Thermo Fisher Scientific:
Rabbit polyclonal anti-ZO-1 #61-7300, dilution 1:50 (IF)
Mouse monoclonal anti-ZO-1 #33-9100, dilution 1:1000 (WB), 1:100 (IF)
Rabbit polyclonal anti-ZONAB #40-2800, dilution 1:250 (WB)
Rabbit monoclonal anti-Claudin-5 #MA5-32614, dilution 1:1000 (WB)

Primary antibodies from New England Biolabs (Cell Signaling):
Mouse mococlona anti-BrdU #5292, dilution 1:100 (IF)
Rabbit polyclonal anti-ZO-2 #2847, dilution 1:1000 (WB)
Rabbit monoclonal anti-MYC-Tag #2278S, dilution 1:1000 (WB)
Rabbit monoclonal anti-phospho-YB1 (Ser102) #2900S, dilution 1:1000 (WB)
Rabbit monoclonal anti-caspase-3 #9665T (discontinued), dilution 1:1000 (WB)
Mouse monoclonal anti-β-Actin #3700S, dilution 1:10 000 (WB)
Rabbit polyclonal anti-YB1 (D299) #4202S, dilution 1:25 (IF)
Rabbit monoclonal anti-YB1 (D2A11) #9744S, dilution 1:1000 (WB)

Primary antibodies from BD Biosciences:
Mouse monoclonal anti-G3BP1 #611126, dilution 1:1000 (WB), 1:100(IF)
Mouse monoclonal anti-β-catenin #610153, dilution 1:1000 (WB)
Mouse monoclonal anti-γ-catenin (JUP) #610253, dilution 1:1000 (WB)

Primary antibody from R&D Systems:
Goat polyclonal anti-VE-cadherin #AF938, dilution 1:1000 (WB)

Primary antibody from Santa Cruz:
Mouse monoclonal anti-EMAP II/AIMP1 #sc-393228, dilution 1:1000 (WB)
Mouse monoclonal anti-FUS/TLS #sc-47711, dilution 1:1000 (WB)
Mouse monoclonal anti-Ribosomal Protein L23a #sc-517097, dilution 1:1000 (WB)

Secondary antibodies for immunoblots from Jackson ImmunoResearch Laboratories:
Peroxidase AffiniPure Goat Anti-Mouse IgG (H+L) #115-035-146, dilution 1:5000
Peroxidase AffiniPure Donkey Anti-Rabbit IgG (H+L) #711-035-152, dilution 1:5000
Peroxidase AffiniPure Donkey Anti-Goat IgG (H+L) #705-035-003, dilution 1:5000

Secondary antibodies from Thermo Fisher Scientific:
Alexa Fluor 488-conjugated Goat anti-Rabbit IgG (H+L) #A-11008, dilution 1:100
Alexa Fluor 488-conjugated Goat anti-Mouse IgG (H+L) #A-11001, dilution 1:100
Alexa Fluor 488-conjugated F(ab')2-Goat anti-Mouse IgG (H+L) #A48286TR, dilution 1:100
Alexa Fluor 488-conjugated Donkey anti-Goat IgG (H+L) #A11055, dilution 1:100
Alexa Fluor 488-higly cross-adsorbed Donkey anti-Rabbit IgG (H+L) #A21206, dilution 1:100
Alexa Fluor 568-higly cross-adsorbed Donkey anti-Rabbit IgG (H+L) #A10042, dilution 1:100
Alexa Fluor 568-higly cross-adsorbed Donkey anti-Mouse IgG (H+L) #A10037, dilution 1:100
Alexa Fluor 647-higly cross-adsorbed Donkey anti-Rabbit IgG (H+L) #A31573, dilution 1:100

Validation

All the antibodies used were commercially developed and validated by the companies
ZO-1 rabbit: validated for Western Blot and Immunofluorescence by the company on MDCK and CACO-2 cell lines and by us on HUVECs and BAECs using siRNA against ZO-1 (PMID: 26846344). https://www.thermofisher.com/antibody/product/ZO-1-Antibody-Polyclonal/61-7300
ZO-1 mouse: validated for Western Blot and Immunofluorescence by the company on MDCK and CACO-2 cell lines and by us on HUVECs and BAECs using siRNA against ZO-1 (PMID: 26846344), and on MLEC from WT and ZO-1 Knock-Out mice within the paper (Fig. 2b, Supplementary Fig. 2a, S2c, S2e, S2h, S2j, Supplementary Fig. 5b). https://www.thermofisher.com/antibody/product/ZO-1-Antibody-clone-ZO1-1A12-Monoclonal/33-9100
ZO-2 Rabbit : validated for Western Blot and Immunofluorescence by the company on 293, COS, A431, NIH/3T3, C6 and PC-12 and by us on BAECs using siRNA against ZO-2 (Supplementary Fig. 2j). https://www.cellsignal.com/product/productDetail.jsp?

productId=2847&country=CA
ZONAB rabbit: validated for Western Blot and Immunofluorescence by the company on mouse cells. https://www.thermofisher.com/antibody/product/ZONAB-Antibody-Polyclonal/40-2800
Claudin-5 Rabbit : validated for Western Blot and Immunofluorescence by the company on human cells nd by us on HUVECs using siRNA against Claudin-5 (Supplementary Fig. 2i). https://www.thermofisher.com/antibody/product/Claudin-5-Antibody-clone-JM11-22-Recombinant-Monoclonal/MA5-32614
BrdU: validated for Immunofluorescence by the company on HeLa cells and Jurkat cells, incorporated with BrdU. https://www.cellsignal.com/products/primary-antibodies/brdu-bu20a-mouse-mab/5292
MYC-Tag: validated for Western Blot and Immunofluorescence by the company on untransfected control cells and transfected cells overexpressing Myc-Bcl-2 and by us on untransfected control BAEC and transfected BAECs overexpressing Myc-ZO-1 (Fig. 2c, upplementary Fig. 4b). https://www.cellsignal.com/product/productDetail.jsp?productId=2278&country=CA
phospho-YB1 (Ser102): validated for Western Blot by the company on MCF-7 cells line treated or not by IGF-1. https://www.cellsignal.com/product/productDetail.jsp?productId=2900&country=CA
caspase-3: validated for Western Blot by the company on HeLa and NIH/3T3 cell lines. https://www.cellsignal.com/product/productDetail.jsp?productId=9665
β-Actin: validated for Western Blot by the company on COS, HeLa, C2C12, C6 and CHO cell lines. https://www.cellsignal.com/product/productDetail.jsp?productId=3700&country=CA. Routinely used in the laboratory.
G3BP1: validated for Western Blot and Immunofluorescence by the company on SW-13 and A431 cell lines. https://www.bdbiosciences.com/en-ca/products/reagents/microscopy-imaging-reagents/immunofluorescence-reagents/purified-mouse-anti-human-g3bp.611126
β-catenin: validated for Western Blot and Immunofluorescence by the company on HeLa and A431 cell lines and by us on HUVECs and BAECs using siRNA against β-catenin (PMID: 22936663, 28320874) (Supplementary Fig. 2h, Supplementary Fig. 3a). https://www.bdbiosciences.com/en-ca/products/reagents/microscopy-imaging-reagents/immunofluorescence-reagents/purified-mouse-anti-catenin.610153
γ-catenin (JUP): validated for Western Blot and Immunofluorescence by the company on MCF-1 cell line and by us on BAECs using siRNA against JUP (PMID: 26846344). https://www.bdbiosciences.com/en-ca/products/reagents/microscopy-imaging-reagents/immunofluorescence-reagents/purified-mouse-anti-catenin.610253
VE-cadherin: validated for Western Blot and Immunofluorescence by the company on HUVEC cell line and by us on HUVECs and BAECs using siRNA (PMID: 26846344) and within the paper (Supplementary Fig. 2h, Supplementary Fig. 3a). https://www.rndsystems.com/products/human-ve-cadherin-antibody_af938
EMAP II/AIMP1: validated for Western Blot and Immunofluorescence by the company on THP-1, T98G, WEHI-231, L6 and A-431 cell line. https://www.scbt.com/p/emap-ii-antibody-a-4
FUS/TLS: c4 (A), validated for Western Blot and Immunofluorescence by the company on NAMALWA, RAW 264.7, THP-1, K-562, Jurkat HeLa cell lines. https://www.scbt.com/p/fus-tls-antibody-4h11
Ribosomal Protein L23a (RPL23A). validated for Western Blot and Immunofluorescence by the company on HeLa cell lines. https://www.scbt.com/p/ribosomal-protein-l23a-antibody-3e11?requestFrom=search
YB1 (D2A11). validated for Western Blot by the company on MCF-7, C2C12, H-4-II-E and COS-7 cell lines and by us on HUVECs and BAECs by siRNA against YB1 (fig. 5e, Supplementary Fig. 4a). https://www.cellsignal.com/product/productDetail.jsp?productId=9744&country=CA
YB1 (D299): validated for Western Blot by the company on MCF-7, NIH/3T3, C6 and COS cell lines and by us on HUVECs and BAECs by siRNA against YB1 (Fig. 5d). https://www.cellsignal.com/products/primary-antibodies/yb1-d299-antibody/4202?site-search-type=Products&N=4294956287&Ntt=+%234202s&fromPage=plp&_requestid=3131750
Peroxidase AffiniPure Goat Anti-Mouse IgG (H+L): Validated for use in for Western blotting by company. https://www.jacksonimmuno.com/catalog/products/115-035-146
Peroxidase AffiniPure Donkey Anti-Rabbit IgG (H+L): Validated for use in for Western blotting by company. https://www.jacksonimmuno.com/catalog/products/711-035-152
Peroxidase AffiniPure Donkey Anti-Goat IgG (H+L): Validated for use in for Western blotting by company. https://www.jacksonimmuno.com/catalog/products/705-035-003
Alexa Fluor 488-conjugated Goat anti-Rabbit IgG (H+L). Validated for use in for immunofluorescence by company. https://www.thermofisher.com/antibody/product/Goat-anti-Rabbit-IgG-H-L-Cross-Adsorbed-Secondary-Antibody-Polyclonal/A-11008
Alexa Fluor 488-conjugated Goat anti-Mouse IgG (H+L). Validated for use in for immunofluorescence by company. https://www.thermofisher.com/antibody/product/Goat-anti-Mouse-IgG-H-L-Cross-Adsorbed-Secondary-Antibody-Polyclonal/A-11001
Alexa Fluor 488-conjugated F(ab')2-Goat anti-Mouse IgG (H+L). Validated for use in for immunofluorescence by company. https://www.thermofisher.com/antibody/product/Goat-anti-Mouse-IgG-H-L-Cross-Adsorbed-Secondary-Antibody-Polyclonal/A48286TR
Alexa Fluor 488-conjugated Donkey anti-Goat IgG (H+L). Validated for use in for immunofluorescence by company. https://www.thermofisher.com/antibody/product/Donkey-anti-Goat-IgG-H-L-Cross-Adsorbed-Secondary-Antibody-Polyclonal/A-11055
Alexa Fluor 488-conjugated Donkey anti-Rabbit IgG (H+L). Validated for use in for immunofluorescence by company. https://www.thermofisher.com/antibody/product/Donkey-anti-Rabbit-IgG-H-L-Highly-Cross-Adsorbed-Secondary-Antibody-Polyclonal/A-21206
Alexa Fluor 568-conjugated Donkey anti-Rabbit IgG (H+L). Validated for use in for immunofluorescence by company. https://www.thermofisher.com/antibody/product/Donkey-anti-Rabbit-IgG-H-L-Highly-Cross-Adsorbed-Secondary-Antibody-Polyclonal/A10042
Alexa Fluor 568-conjugated Donkey anti-Mouse IgG (H+L). Validated for use in for immunofluorescence by company. https://www.thermofisher.com/antibody/product/Donkey-anti-Mouse-IgG-H-L-Highly-Cross-Adsorbed-Secondary-Antibody-Polyclonal/A10037
Alexa Fluor 647-higly cross-adsorbed Donkey anti-Rabbit IgG (H+L). Validated for use in for immunofluorescence by company. https://www.thermofisher.com/antibody/product/Donkey-anti-Rabbit-IgG-H-L-Highly-Cross-Adsorbed-Secondary-Antibody-Polyclonal/A-31573

Eukaryotic cell lines

Policy information about cell lines and Sex and Gender in Research

Cell line source(s)	BAECs and HUVECs were obtained from VEC Technologies (Rensselaer, NY, USA). TeloHAEC (CRL-4052) and HepG2 (HB-8065) were from ATCC (Manassas, VA, USA).

Authentication	BAECs and HUVECs were isolated and authenticated by VEC Technologies. Telo-HAEC and HepG2 were isolated and authenticated by ATCC (STR profiling).
Mycoplasma contamination	Each lot of cells obtained from VEC Technologies are confirmed negatived for mycoplasma infection by the company.
Commonly misidentified lines (See ICLAC register)	No commonly misidentified cell lines were used.

Animals and other research organisms

Policy information about studies involving animals; ARRIVE guidelines recommended for reporting animal research, and Sex and Gender in Research

Laboratory animals	Mouse Strains: C57BL/6J, Tjp1tm2a(KOMP)Wtsi (MGI:98759) C57BL/6N, B6.Cg-Tg(ACTFLPe)9205Dym/J (MGI:2448985) and B6-Tjp1tm2a were used to generate Pdgfb-iCreER;Tjp1fl/fl and Tjpfl/fl mice. Males and females were used at post-natal day7. Retinal vascular developement was examined at post-natal day 7. All animals were housed under controlled conditions with an ambient temperature set at 22°C and relative humidity ranging from 35% to 50% under a 12/12-h light/dark cycle with unrestricted access to food and water throughout the duration of the experiment.
Wild animals	The study did not involve wild animals
Reporting on sex	Sex was not considered as a variable in this study, however equal number of males and females were used throughout. We did not perform intersex statistical analysis.
Field-collected samples	The study did not involve samples collected from the field.
Ethics oversight	All animal studies were approved by the Animal Care Committee of the University of Montreal in agreement with the guidelines established by the Canadian Council on Animal Care.

Note that full information on the approval of the study protocol must also be provided in the manuscript.

Plants

Seed stocks	n/a
Novel plant genotypes	n/a
Authentication	n/a
